# CD24 cross-linking induces apoptosis in, and inhibits migration of, MCF-7 breast cancer cells

**DOI:** 10.1186/1471-2407-8-118

**Published:** 2008-04-24

**Authors:** Jong Bin Kim, Eunyoung Ko, Wonshik Han, Jeong Eon Lee, Kyung-Min Lee, Incheol Shin, Sangmin Kim, Jong Won Lee, Jihyoung Cho, Ji-Yeon Bae, Hyeon-Gun Jee, Dong-Young Noh

**Affiliations:** 1 Cancer Research Institute, Seoul National University College of Medicine, 28 Yongon-dong, Chongno-gu, Seoul 110-744, Korea; 2 Department of Surgery, Seoul National University College of Medicine, 28 Yongon-dong, Chongno-gu, Seoul 110-744, Korea; 3 Department of Life Science, College of Natural Sciences, Hanyang University, 17 Haengdang-dong, Sungdong-gu, Seoul 133-791, Korea; 4 Clinical Research Institute, Seoul National University College of Medicine, 28 Yongon-dong, Chongno-gu, Seoul 110-744, Korea

## Abstract

**Background:**

The biological effects of CD24 (FL-80) cross-linking on breast cancer cells have not yet been established. We examined the impact of CD24 cross-linking on human breast cancer cell line MCF-7.

**Methods:**

MCF-7 and MDA-MB-231 cells were treated with anti-rabbit polyclonal IgG or anti-human CD24 rabbit polyclonal antibodies to induce cross-linking, and then growth was studied. Changes in cell characteristics such as cell cycle modulation, cell death, survival in three-dimensional cultures, adhesion, and migration ability were assayed after CD24 cross-linking in MCF-7.

**Results:**

Expression of CD24 was analyzed by flow cytometry in MDA-MB-231 and MCF-7 cells where 2% and 66% expression frequencies were observed, respectively. CD24 cross-linking resulted in time-dependent proliferation reduction in MCF-7 cells, but no reduction in MDA-MB-231 cells. MCF-7 cell survival was reduced by 15% in three-dimensional culture after CD24 cross-linking. Increased MCF-7 cell apoptosis was observed after CD24 cross-linking, but no cell cycle arrest was observed in that condition. The migration capacity of MCF-7 cells was diminished by 30% after CD24 cross-linking.

**Conclusion:**

Our results showed that CD24 cross-linking induced apoptosis and inhibited migration in MCF-7 breast cancer cells. We conclude that CD24 may be considered as a novel therapeutic target for breast cancer.

## Background

CD24 is expressed in hematopoietic cell types, including B-cell precursors and neutrophils [1], and is also conventionally used as a differentiation marker for keratinocytes [2]. Accumulating evidence supports a role for CD24 in a variety of malignancies, including B-cell lymphoma, renal cell carcinoma, small-cell and non small-cell lung carcinoma, nasopharyngeal carcinoma, hepatocellular carcinoma, bladder carcinoma, epithelial ovarian cancer and breast cancer [3].

CD24, designated 'heat-stable antigen' (HSA) in mice, is a glycosylated cell-surface protein linked to the membrane via a glycosyl-phosphatidylinositol (GPI) anchor [4]. CD24 has several potential N- and O-linked glycosylation sites, which act as ligands for P-selectin [4].

CD24 is involved in cellular adhesion processes and signalling pathways in cancer cells that are dependent on interactions with P-selectin [4]. Moreover, CD24-mediated binding to P-selectin on endothelial cells and platelets may facilitate the exit of tumor cells from the bloodstream and potentiate metastasis [3]. In P-selectin-deficient mice, diminished tumor growth and metastasis is observed, compared with wild-type animals [5]. Moreover, CD24 over-expression is associated with invasiveness in urothelial carcinoma [6] and with migration and invasion in gliomas [7]. These studies collectively imply that CD24 might play an important role in tumorigenesis and in the progression of cancer. Moreover, CD24 expression is suggested to be a marker of poor prognosis in various cancers, including breast carcinoma [8]. In breast cancer, CD24 mediates progression, metastasis, and rolling of tumor cells through interactions with P-selectin [9]. Additionally, CD24 function may be related to tamoxifen resistance [10]. In this study, MCF-7 cells were used as a general breast cancer cell model, based on the fact that these cells are derived from a pleural effusion from a patient with metastatic breast carcinoma [11]. MCF-7 cells are adherent, and they aggregate into clusters under standard culture conditions to form duct like structures that mimic luminal structures observed in under three-dimensional culture conditions [12]. For this reason, MCF-7 cells have primarily been used as model of the luminal breast cancer cell type, which express CK8/18 [13], CK19 [14], CD24 [15] and the estrogen receptor [16], but not vimentin [11].

Recently, CD24 was recommended as a prognostic indicator of poor patient survival in breast cancer [17]. It is known that CD24 mRNA becomes unregulated after amino acid starvation in MCF-7 cells and that the CD24 protein is expressed more than 80% in MCF-7 cells [15]. For this reason it is suggested that CD24 may play an important role in the progression and metastasis of human breast cancer [8,18].

The aim of this study was to further clarify the role of CD24 in breast cancer cell growth using a cross-linking approach. Changes in viable cell number, on adhesion and migration abilities, and in cell growth and death were assessed. We did to study directly impact on cross-linking with CD24 (FL-80) antibody in MCF-7 human breast cancer cell line.

## Methods

### Cell culture

Unless otherwise specified, all reagents were purchased from Sigma (St. Louis, MO). MDA-MB-231 and MCF-7 cell lines were cultured in Dulbecco's modified Eagle's medium (DMEM) containing 10% fetal bovine serum (FBS). For the anchorage-dependent culture, 5×10^5 ^cells were seeded on a tissue culture dish (Falcon, San Jose, CA). Cells were incubated at 37°C in a humidified atmosphere containing 5% CO^2^. Photographs were obtained with an inverted system microscope (IX51 model) equipped with a DP50 camera system (Olympus, Tokyo, Japan).

### CD24 expression in breast cell lines

To detach MDA-MB-231 and MCF-7 cells, cultures were trypsinized for 3 min to minimize growth inhibition, and detachment was monitored with a phase-contrast microscope. After visual identification of detached cells, 5 ml of medium containing serum was added to the culture for trypsin inactivation. Cells were collected by centrifugation, and washed with 5 ml of PBS. Following a second centrifugation step, pellets were resuspended in 500 μl total volume with the appropriate amounts of antibodies and supplement of PBS. The PE anti-human CD24 antibody (BD Pharmingen, NJ, USA) was applied according to the manufacturer's manual. After antibody binding for 25 min at room temperature, cells were rinsed three times with PBS and flow cytometry analyses were performed in triplicate using a FACSCalibur system (Becton & Dickinson, San Jose, CA).

### Assessment of changes in proliferation of MDA-MB-231 and MCF-7 cells upon CD24 cross-linking

For each line, 5×10^5 ^cells were seeded with DMEM containing 10% FBS and after 24 h, they were washed twice with PBS. Fresh DMEM+10% FBS was added to the dish, and CD24 was cross-linked with anti-rabbit polyclonal IgG antibody and anti-human CD24 (FL-80) rabbit polyclonal antibody (Santa Cruz Biotechnology, Franklin Lakes, NJ). Rabbit-polyclonal anti-CD24 (FL-80) antibody was raised against amino acids 1–80, representing full length CD24 of human origin. For each condition, the number of viable cells was estimated by trypan blue staining.

### Three-dimensional matrigel culture, and MTT assays

For a three-dimensional culture, a millicell (millipore, Billerica, MA, USA) of 3 μm pore size was coated with 5 mg/ml matrigel (BD Pharmingen, NJ, USA) on a 6-well plate at 37°C for 1 h. MCF-7 cells were trypsinized and seeded onto millicells in a dose dependent manner of 1×10^5^, 3×10^5^, or 5×10^5 ^cells at 37°C for 24 h. After 24 h, cells cross-linked with anti-rabbit polyclonal IgG (500 ng/ml) or anti-human CD24 (FL-80) rabbit polyclonal antibody (500 ng/ml) at 37°C for 72 h, then the media was removed. 100 μl of MTT reagents (5 mg/ml) were added to each millicell, whch was incubated at 37°C for 3 h and the media was removed. 200 μl of dimethylsulphoxide (DMSO) was then added to each millicell with shaking for 10 min and the absorbance was measured spectrophotometrically at 595 nm.

### Cell cycle analysis and Annexin V staining

After cross-linking for 72 h, the MCF-7 culture was harvested, where cells were fixed in 70% ethanol for 1 h, and washed with PBS. Cells were then treated with 100 μg/ml RNase A for 1 h at 37°C, followed by 25 μg/ml propidium iodide solution. Flow cytometry was then performed in triplicate for each experiment on a FACSCalibur system (Becton & Dickinson, San Jose, CA). Annexin V staining was done according to the manufacturer's protocol (BD Pharmingen, NJ, USA). MCF-7 cells were washed with PBS and trypsinized. After centrifugation, cells were washed twice with cold PBS, and resuspended in binding buffer (10 mM HEPES, pH 7.4, 150 mM NaCl, 5 mM KCl, 1 mM MgCl_2_, 1.8 mM CaCl_2_) at a concentration of 1×10^6 ^cells/ml. An aliquot (100 μl) of the solution containing 1 × 10^5 ^cells was transferred to a 5 ml culture tube, and 5 μl each of Annexin V-FITC and PI were added. After vortexing, cells were incubated for 15 min at room temperature (25°C) in the dark, and 400 μl of 1× binding buffer was added to each tube. Flow cytometry was performed on a FACSCalibur system (Becton & Dickinson, San Jose, CA) within 1 h.

### Adhesion assay

For adhesion assay, a 96-well plate was coated with type I collagen (10 μg/ml) at 37°C for 1 h, washed twice with PBS, and blocked with 10% FBS in PBS. MCF-7 cells were seeded and incubated with anti-rabbit polyclonal IgG (500 ng/ml) or anti-human CD24 rabbit polyclonal antibody (500 ng/ml) at 37°C for 24 h. After 24 h, MCF-7 cells were washed twice with PBS and trypsinized, then seeded at 2×10^4^/well and incubated at 37°C incubator for 24 h. Adherent cells were fixed with 3.7% paraformaldehyde at room temperature for 20 min, and cells were air-dried for 5 min, followed by staining with 0.1% crystal violet in methanol at room temperature for 45 min. Cells were washed three times with PBS, and rinsed with 0.1 M sodium citrate at room temperature for 30 min. The absorbance of the resulting solution was measured at 595 nm.

### Migration assay

The migration assay was performed following the manufacturer's protocol (BD Pharmingen, NJ, USA). MCF-7 cells were pre-treated with anti-rabbit polyclonal IgG (500 ng/ml) or anti-human CD24 rabbit polyclonal antibody (500 ng/ml) at 37°C for 24 h. 2×10^4^/well cells were seeded onto the upper side of a transwell chamber (BD Pharmingen, NJ, USA) and incubated at 37°C for 24 h. Cells from the upper side of the transwell were then scraped off mechanically, fixed with 100% methanol at room temperature for 2 min and stained with 1% toluidine blue in 1% borax solution at room temperature for 2 min and then counted with an inverted system microscope.

### Statistical analysis

All data were compiled from a minimum of three replicate experiments. Data for statistical analysis were expressed as the mean ± standard error. Comparison of results from treated versus control cells was done using *t*-stests. *p *values of less than 0.05 were considered statistically significant.

## Results

### CD24 expression in MDA-MB-231 and MCF-7 cells

**Figure 1:**
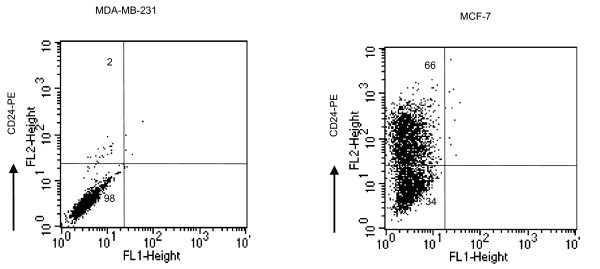
**CD24 expression in MDA-MB-231 and MCF-7 cells by FACS (%)**. MDA-MB-231 and MCF-7 cells were cultured in DMEM containing 10% FBS for 72 h. Flow cytometry analysis was performed with PE anti-human CD24 antibody on a FACSCalibur system. A representative result is shown.

Expression of CD24 was analyzed in MDA-MB-231 and MCF-7 cells by flow cytometry, which revealed that 2% of MDA-MB-231 cells and 66% of MCF-7 cells expressed CD24 (Fig. 1). Based on this result, we selected MDA-MB-231 as negative control and MCF-7 as an experimental cell line for examining the effect of CD24 cross-linking on the growth of breast cancer cells.

### Proliferation of control and MCF-7 cells upon CD24 cross-linking

**Figure 2:**
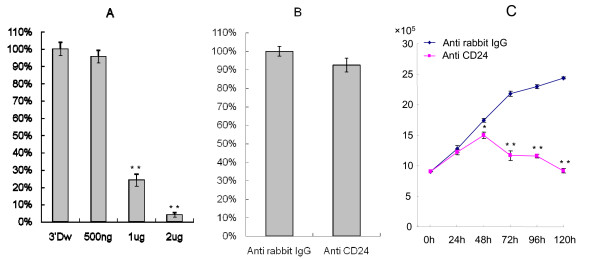
**Viability of MDA-MB-231 and MCF-7 cells after CD24 cross-linking**. A) MCF-7 cross-linked with anti-rabbit polyclonal IgG in a dose dependent manner for 72 h. B) MDA-MB-231 cells were cross-linked with 500 ng/ml anti-rabbit polyclonal IgG or anti-human CD24 rabbit polyclonal antibody for 72 h. C). MCF-7 cells were cross-linked with 500 ng/ml anti-rabbit polyclonal IgG antibody or anti-human CD24 rabbit polyclonal antibody in a time dependent manner for 120 h. A-B) Relative cell survival rate is shown as percent survival versus control cells after cross-linking with anti-rabbit polyclonal IgG. C) MCF-7 cell survival is shown versus control cell survival when CD24 was cross-linked with anti-rabbit polyclonal IgG. Data represent means of at least three independent experiments and standard errors of the means. *, *p *value of less than 0.05 **, *p *value of less than 0.01.

72 h after cross-linking, the number of viable cells was counted to analyze the effects of cross-linking on cell growth. MCF-7 cell viability did not change after cross-linking with 500 ng/ml anti-rabbit polyclonal IgG. However, cell viability after 72 h was reduced by 75% and 96% at antibody concentrations of 1 μg/ml and 2 μg/ml, respectively (Fig. 2A). We also examined cell growth in five different culture media: culture medium without distilled water, culture medium with 0.025% distilled water, distilled water with 0.00025% sodium azide, distilled water with 0.00025% gelatin, and distilled water with 0.00025% PBS. We did not find difference in cell growth in each culture condition (Data not shown). Accordingly, 500 ng/ml was selected as the dose of anti-rabbit polyclonal IgG that exerted no effect on viability. MDA-MB-231 and MCF-7 cells were treated with 500 ng/ml anti-rabbit polyclonal IgG or 500 ng/ml anti-human CD24 rabbit polyclonal antibody. There was no change in MDA-MB-231 cell growth (Fig. 2B). In contrast, MCF-7 cell growth decreased by 4%, 14%, 46%, 50%, and 62% at time-points of 24 h, 48 h, 72 h, 96 h, and 120 h, respectively (Fig. 2C).

### Inhibition of growth after CD24 cross-linking in three-dimensional culture

**Figure 3:**
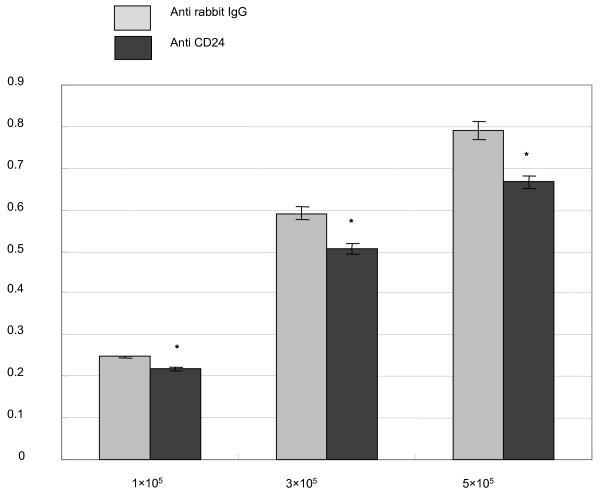
**MCF-7 cell growth in three-dimensional culture**. 1×10^5^, 3×10^5^, and 5×10^5 ^cells were cultured in DMEM containing 10% FBS in the three-dimensional matrigel (5 mg/ml) for 24 h and then CD24 was cross-linked with 500 ng/ml anti-rabbit polyclonal IgG or anti-human CD24 rabbit polyclonal antibody for 72 h. Cell viability in three-dimensional culture was examined by MTT assay. The data are shown as the absorbance of MCF-7 survival versus the control cell survival after CD24 cross-linking with anti-rabbit polyclonal IgG. Means of at least three independent experiments are compared and standard errors are shown. *, *p *value of less than 0.05.

Three-dimensional culture using matrigel was predicted to be a useful method for confirming the effect of cross-linking *in vitro*. We examined growth inhibition after CD24 cross-linking using matrigel containing ECM. MCF-7 cells were cross-linked with anti-rabbit polyclonal IgG and anti-human CD24 rabbit polyclonal antibody at the 500 ng/ml dosage for 72 h. When cultured on top of three-dimensional matrigel, MCF-7 cells cross-linked with anti-human CD24 rabbit polyclonal antibody decreased 15% compared to cells cross-linked with anti-rabbit polyclonal IgG (Fig. 3).

### Cell cycle and apoptosis analyses after CD24 cross-linking

**Figure 4:**
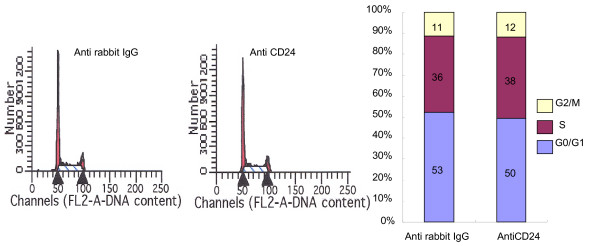
**Changes in cell cycle after CD24 cross-linking 72 h**. The cell cycle was analyzed by FACS after DNA staining with propidium iodide and a representative experiment (from three total) is shown.

**Figure 5:**
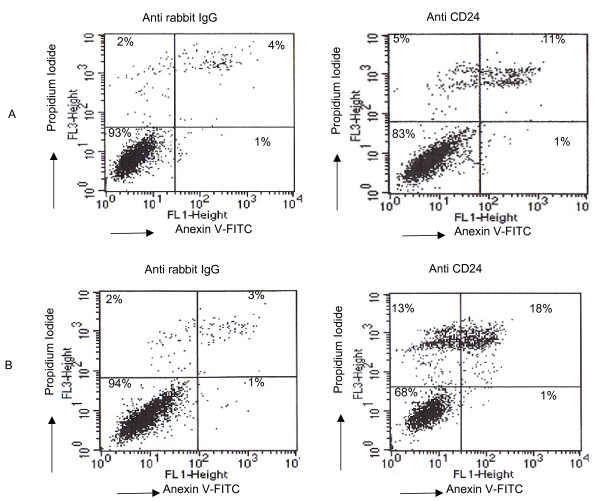
**Changes in apoptosis after CD24 cross-linking**. A) Cross-linking for 72 h. B) Cross-linking for 96 h. Cells were stained with FITC-conjugated annexin V in a buffer containing propidium iodide and analyzed by flow cytometry. For each group of cells, the percentage of survival is shown in the lower left quadrant, where both in annexin V and propidium iodide levels are low. One of three representative experiments is presented.

We observed a decrease in cell number after CD24 cross-linking. To determine whether the cause of decreased cell number was cell cycle arrest or apoptosis, we analyzed DNA content with propidium iodide solution and annexin V by flow cytometry. Stained DNA contents after CD24 cross-linking were similar in each cell type compared to after cross-linking with anti-rabbit polyclonal IgG (Fig. 4). In the apoptosis assay, cell population decreased by 10% and 26% at 72 h and 96 h, respectively, exhibiting increased apoptosis after CD24 cross-linking (Fig. 5A,B).

### Effects of CD24 cross-linking on MCF-7 cell adhesion and migration

**Figure 6:**
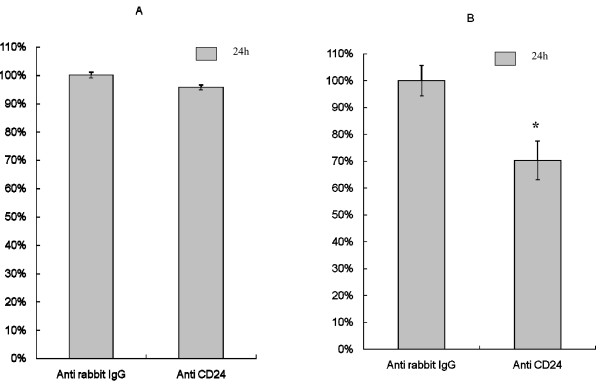
**Effects of CD24 cross-linking on the adhesion and migration capacity of MCF-7 cells**. A) Relative adhesion rate in the data is shown as percent adherent cells versus control cells where CD24 was cross-linked with anti-rabbit polyclonal IgG. B) Relative migrating cell rate is shown as percent migrating cells versus control cells where CD24 was cross-linked with anti-rabbit polyclonal IgG. *, *p *value of less than 0.05.

We assumed that CD24 was a cell adhesion molecule in MCF-7 cells. MCF-7 was pre-treated with anti-rabbit polyclonal IgG or anti-human CD24 rabbit polyclonal antibody (500 ng/ml) for 24 h. The adhesion capacity of harvested MCF-7 cells after CD24 cross-linking was not significantly reduced (Fig. 6A), but the number of migrating cells was reduced by 30% (Fig. 6B).

## Discussion

CD24 is important for progression, migration, and metastasis of human breast cancer [8]. However, the specific functions of CD24 in breast cancer are unclear. Here, we showed that CD24 cross-linking is sufficient to inhibit tumor growth (Fig. 2C), and migration (Fig. 6B) in MCF-7 breast cancer cells. Additionally, apoptosis increased upon CD24 cross-linking (Fig. 5).

Our results are in agreement with previous findings when Suzuki *et al*. [19] reported that CD24 cross-linking induced apoptosis in a human B-cell subset during the early activation stage through interactions with glycolipid-enriched membrane domains. Taguchi et al. [20] demonstrated that apoptosis observed upon cross-linking CD24 did not result from non-specific binding of either mouse immunoglobulin or secondary rabbit polyclonal anti-mouse immunoglobulin antibody, but from treatment with a combination of anti-CD24 and rabbit anti-mouse immunoglobulin antibodies in KM-3 pro-B cells. To confirm that the apoptosis observed upon cross-linking of CD24 was not resulting from the non-specific binding of secondary rabbit polyclonal antibodies, MCF-7 cells were cross-linked with anti-human CD24 (SN3) mouse monoclonal antibody raised against NALM-1 human pre-B leukemia cell line. Effect of cross-linking with anti-human CD24 (SN3) mouse monoclonal antibody was similar to what was observed anti-human CD24 (FL-80) rabbit polyclonal antibody (Additional files. 2, 3). Several analogous reports documented proliferation and apoptosis in human B-cells [19] and murine thymocytes [21] after CD24 cross-linking.

In contrast to our findings, Schabath and colleagues showed that MDA-MB-231 cells (used as a control in this study) transfected to express CD24 had reduced migration and tumor growth in NOD/SCID mice [22]. We cross-linked CD24 in MCF-10A breast cells expressing CD24 and observed inhibited growth in the CD24 expressing cells (Additional files. 1), similar to what was observed in MCF-7 cells.

We found that CD24 cross-linking exhibited an inhibitory effect on breast cancer cell growth in the three-dimensional culture system. In agreement with our findings, Wang *et al*. [23] reported a reduction in T-cell proliferation upon blockage with an anti-HSA antibody. Moreover, cross-linking of CD24 induced apoptosis in murine thymocytes [21]. Jung and colleagues [21] demonstrated that apoptosis triggered by CD24 cross-linking results in the generation of reactive oxygen species (ROS), and that the release of apoptosis inducing factor (AIF) does not lead to caspase activation in murine thymocytes.

Migration capacities of MCF-7 cells were reduced by 30% after CD24 cross-linking. Our results further indicate that CD24 is involved in migration in MCF-7 cells, and imply that tumor progression can be inhibited by CD24 cross-linking (Fig. 6B). Using *in vitro *migration assays (matrigel) and *in vivo *immunohistochemical staining, Senner *et al*. [7] have found, in a rat model, that CD24-positive gliomas are more aggressive than CD24 negative implants, but did not observe a greater migration rate of CD24-positive cells in matrigel assays, which is in concordance with our data. CD24 is an adhesion molecule [2] and during tumor progression, adhesion to the extracellular matrix (ECM) is the initial step for invasion and metastasis [24]. In our study the adhesion of MCF-7 cells was reduced slightly, but not significantly after CD24 cross-linking.

Interestingly, we observed variable expression rates of CD24 in both control and MCF-7 cells. Figure 1 depicts CD24 expression in 2% of control cells and 66% of MCF-7 cells. However, expression levels of CD24 in MCF-7 cells ranged from 66% to 98%, depending on the passage number and culture conditions, including culture medium serum composition (data not shown). A number of reports show that alterations in protein expression are dependent on the culture environment. For instance, serum in MCF-10A cell culture plays an important role in CD24 expression [11]. Estrogen-receptor expression is altered in MCF-7 and BT474 cells depending on the passage number [25].

Recently, CD44^+^CD24^-/low ^lineage-cells were implicated in breast cancer initiated tumorigenesis in NOD/SCID mice [26]. This work suggested that the tumor initiating cells were cancer stem cells. Furthermore, early cancer cells detected in bone marrow of breast cancer patients were observed to have breast cancer stem sell phenotype [27], but the frequency of CD44^+^/CD24^-/low ^cells in breast cancer tissue was not correlated with clinical outcome [28]. CD24 was suggested as a marker of luminal cells in the breast [29], and a previous report indicated the existence of progenitor cells in luminal epithelia which could be reflective of myoepithelial cells [30]. On the other hand, normal stem cells have been isolated using CD24, CD29, and CD49f in mouse mammary glands [31,32], so a single, definitive breast cancer stem cell marker has yet to be defined.

## Conclusion

In conclusion, we demonstrated that CD24 cross-linking induced apoptosis and inhibited migration ability in MCF-7 human breast cancer cells. It is possible that CD24 *in vivo *may be a novel target for breast cancer treatment.

## Competing interests

The authors declare that they have no competing interests.

## Authors' contributions

All the authors contributed to the conception of the work during the initial stages and study design, analysis and interpretation of the data, as well as drafting and critical revision of the important intellectual content. All authors approved the final version of the manuscript to be published. JBK and EK have equally contributed to all parts of this study. DYN and WH were in charge of the general supervision of the research. The order of authorship was based on a joint decision.

## Pre-publication history

The pre-publication history for this paper can be accessed here:



## Supplementary Material

Additional file 2 **Viability of MCF-7 cross-linked with anti-human CD24 mouse monoclonal antibody**. A) MCF-7 cells were cross-linked with anti-mouse monoclonal IgG antibody in a dose dependent manner for 72 h. B) MCF-7 cells were cross-linked with 500 ng/ml anti-mouse monoclonal IgG antibody or anti-human CD24 mouse monoclonal antibody for 72 h. A-B) Relative survival cell rate is shown as percent survivals versus in treatment versus control cells where CD24 was cross-linked with anti-mouse monoclonal IgG. Data represent means of at least three independent experiments and standard errors of the means. **, *p *value of less than 0.01. Click here for file

Additional file 3 **Changes in apoptosis after CD24 cross-linking with anti-human CD24 mouse monoclonal antibody**. MCF-7 cells were cross-linked with 500 ng/ml anti-mouse monoclonal IgG antibody or anti-human CD24 mouse monoclonal antibody for 72 h. Cells were stained with FITC-conjugated annexin V in a buffer containing propidium iodide and analyzed by flow cytometry. For each group of cells, the percentage of survival is shown in the lower left quadrant, where both in annexin V and propidium iodide levels are low. One of three representative experiments is presented. Click here for file

Additional file 1 **CD24 expression and cell viability after CD24 cross-linking in MCF-10A cells**. A) CD24 expression was analysed with PE anti-human CD24 antibody by flow cytometry on a FACSCalibur system. One of three the representative experiments are shown in the result. B) MCF-10A was cross-linked with 500 ng/ml anti-rabbit polyclonal IgG or anti-human CD24 rabbit polyclonal antibody 72 h. B) Relative survival cell rate is shown as percent survivals versus in treatment versus control cells where CD24 was cross-linked with anti-rabbit polyclonal IgG. C). MCF-10A was treated with 500 ng/ml of anti-rabbit polyclonal IgG or anti-human CD24 rabbit polyclonal antibody in a time dependent manner for 72 h. C) MCF-10A cell survival is shown versus control cell survival after CD24 cross-linking with anti-rabbit polyclonal IgG. Means of at least three independent experiments are presented with standard errors. *, *p *value of less than 0.05 **, *p *value of less than 0.01. Click here for file

## References

[B1] Baumann P, Cremers N, Kroese F, Orend G, Chiquet-Ehrismann R, Uede T, Yagita H, Sleeman JP (2005). CD24 expression causes the acquisition of multiple cellular properties associated with tumor growth and metastasis. Cancer research.

[B2] Magnaldo T, Barrandon Y (1996). CD24 (heat stable antigen, nectadrin), a novel keratinocyte differentiation marker, is preferentially expressed in areas of the hair follicle containing the colony-forming cells. Journal of cell science.

[B3] Kristiansen G, Sammar M, Altevogt P (2004). Tumour biological aspects of CD24, a mucin-like adhesion molecule. J Mol Histol.

[B4] Aigner S, Ruppert M, Hubbe M, Sammar M, Sthoeger Z, Butcher EC, Vestweber D, Altevogt P (1995). Heat stable antigen (mouse CD24) supports myeloid cell binding to endothelial and platelet P-selectin. Int Immunol.

[B5] Friederichs J, Zeller Y, Hafezi-Moghadam A, Grone HJ, Ley K, Altevogt P (2000). The CD24/P-selectin binding pathway initiates lung arrest of human A125 adenocarcinoma cells. Cancer research.

[B6] Choi YL, Lee SH, Kwon GY, Park CK, Han JJ, Choi JS, Choi HY, Kim SH, Shin YK (2007). Overexpression of CD24: association with invasiveness in urothelial carcinoma of the bladder. Archives of pathology & laboratory medicine.

[B7] Senner V, Sturm A, Baur I, Schrell UH, Distel L, Paulus W (1999). CD24 promotes invasion of glioma cells in vivo. J Neuropathol Exp Neurol.

[B8] Kristiansen G, Winzer KJ, Mayordomo E, Bellach J, Schluns K, Denkert C, Dahl E, Pilarsky C, Altevogt P, Guski H (2003). CD24 expression is a new prognostic marker in breast cancer. Clin Cancer Res.

[B9] Aigner S, Ramos CL, Hafezi-Moghadam A, Lawrence MB, Friederichs J, Altevogt P, Ley K (1998). CD24 mediates rolling of breast carcinoma cells on P-selectin. Faseb J.

[B10] Surowiak P, Materna V, Paluchowski P, Matkowski R, Wojnar A, Maciejczyk A, Pudelko M, Kornafel J, Dietel M, Kristiansen G (2006). CD24 expression is specific for tamoxifen-resistant ductal breast cancer cases. Anticancer research.

[B11] Sheridan C, Kishimoto H, Fuchs RK, Mehrotra S, Bhat-Nakshatri P, Turner CH, Goulet R, Badve S, Nakshatri H (2006). CD44+/CD24-breast cancer cells exhibit enhanced invasive properties: an early step necessary for metastasis. Breast Cancer Res.

[B12] Oesterreich S, Zhang P, Guler RL, Sun X, Curran EM, Welshons WV, Osborne CK, Lee AV (2001). Re-expression of estrogen receptor alpha in estrogen receptor alpha-negative MCF-7 cells restores both estrogen and insulin-like growth factor-mediated signaling and growth. Cancer research.

[B13] Long X, Nephew KP (2006). Fulvestrant (ICI 182,780)-dependent interacting proteins mediate immobilization and degradation of estrogen receptor-alpha. The Journal of biological chemistry.

[B14] Charafe-Jauffret E, Ginestier C, Monville F, Finetti P, Adelaide J, Cervera N, Fekairi S, Xerri L, Jacquemier J, Birnbaum D (2006). Gene expression profiling of breast cell lines identifies potential new basal markers. Oncogene.

[B15] Liu W, Vadgama JV (2000). Identification and characterization of amino acid starvation-induced CD24 gene in MCF-7 human breast cancer cells. International journal of oncology.

[B16] Miller MA, Lippman ME, Katzenellenbogen BS (1984). Antiestrogen binding in antiestrogen growth-resistant estrogen-responsive clonal variants of MCF-7 human breast cancer cells. Cancer research.

[B17] Lim SC, Oh SH (2005). The role of CD24 in various human epithelial neoplasias. Pathology, research and practice.

[B18] Fogel M, Friederichs J, Zeller Y, Husar M, Smirnov A, Roitman L, Altevogt P, Sthoeger ZM (1999). CD24 is a marker for human breast carcinoma. Cancer letters.

[B19] Suzuki T, Kiyokawa N, Taguchi T, Sekino T, Katagiri YU, Fujimoto J (2001). CD24 induces apoptosis in human B cells via the glycolipid-enriched membrane domains/rafts-mediated signaling system. J Immunol.

[B20] Taguchi T, Kiyokawa N, Mimori K, Suzuki T, Sekino T, Nakajima H, Saito M, Katagiri YU, Matsuo N, Matsuo Y (2003). Pre-B cell antigen receptor-mediated signal inhibits CD24-induced apoptosis in human pre-B cells. J Immunol.

[B21] Jung KC, Park WS, Kim HJ, Choi EY, Kook MC, Lee HW, Bae Y (2004). TCR-independent and caspase-independent apoptosis of murine thymocytes by CD24 cross-linking. J Immunol.

[B22] Schabath H, Runz S, Joumaa S, Altevogt P (2006). CD24 affects CXCR4 function in pre-B lymphocytes and breast carcinoma cells. Journal of cell science.

[B23] Wang YC, Sashidharamurthy R, Nagarajan S, Selvaraj P (2006). B7-1-HSA (CD80-CD24), a recombinant hybrid costimulatory molecule retains ligand binding and costimulatory functions. Immunology letters.

[B24] Hough MR, Rosten PM, Sexton TL, Kay R, Humphries RK (1994). Mapping of CD24 and homologous sequences to multiple chromosomal loci. Genomics.

[B25] Lostumbo A, Mehta D, Setty S, Nunez R (2006). Flow cytometry: a new approach for the molecular profiling of breast cancer. Exp Mol Pathol.

[B26] Al-Hajj M, Wicha MS, Benito-Hernandez A, Morrison SJ, Clarke MF (2003). Prospective identification of tumorigenic breast cancer cells. Proceedings of the National Academy of Sciences of the United States of America.

[B27] Balic M, Lin H, Young L, Hawes D, Giuliano A, McNamara G, Datar RH, Cote RJ (2006). Most early disseminated cancer cells detected in bone marrow of breast cancer patients have a putative breast cancer stem cell phenotype. Clin Cancer Res.

[B28] Abraham BK, Fritz P, McClellan M, Hauptvogel P, Athelogou M, Brauch H (2005). Prevalence of CD44+/CD24-/low cells in breast cancer may not be associated with clinical outcome but may favor distant metastasis. Clin Cancer Res.

[B29] Sleeman KE, Kendrick H, Ashworth A, Isacke CM, Smalley MJ (2006). CD24 staining of mouse mammary gland cells defines luminal epithelial, myoepithelial/basal and non-epithelial cells. Breast Cancer Res.

[B30] Gudjonsson T, Villadsen R, Nielsen HL, Ronnov-Jessen L, Bissell MJ, Petersen OW (2002). Isolation, immortalization, and characterization of a human breast epithelial cell line with stem cell properties. Genes & development.

[B31] Kordon EC, Smith GH (1998). An entire functional mammary gland may comprise the progeny from a single cell. Development (Cambridge, England).

[B32] Shackleton M, Vaillant F, Simpson KJ, Stingl J, Smyth GK, Asselin-Labat ML, Wu L, Lindeman GJ, Visvader JE (2006). Generation of a functional mammary gland from a single stem cell. Nature.

