# Supplementary figures and images for: CD24 cross-linking induces apoptosis in, and inhibits migration of, MCF-7 breast cancer cells
Source: BMC Cancer. 2008 Apr 24;8:118. doi: 10.1186/1471-2407-8-118 (PMC2386794; doi:10.1186/1471-2407-8-118)

## Slide 1
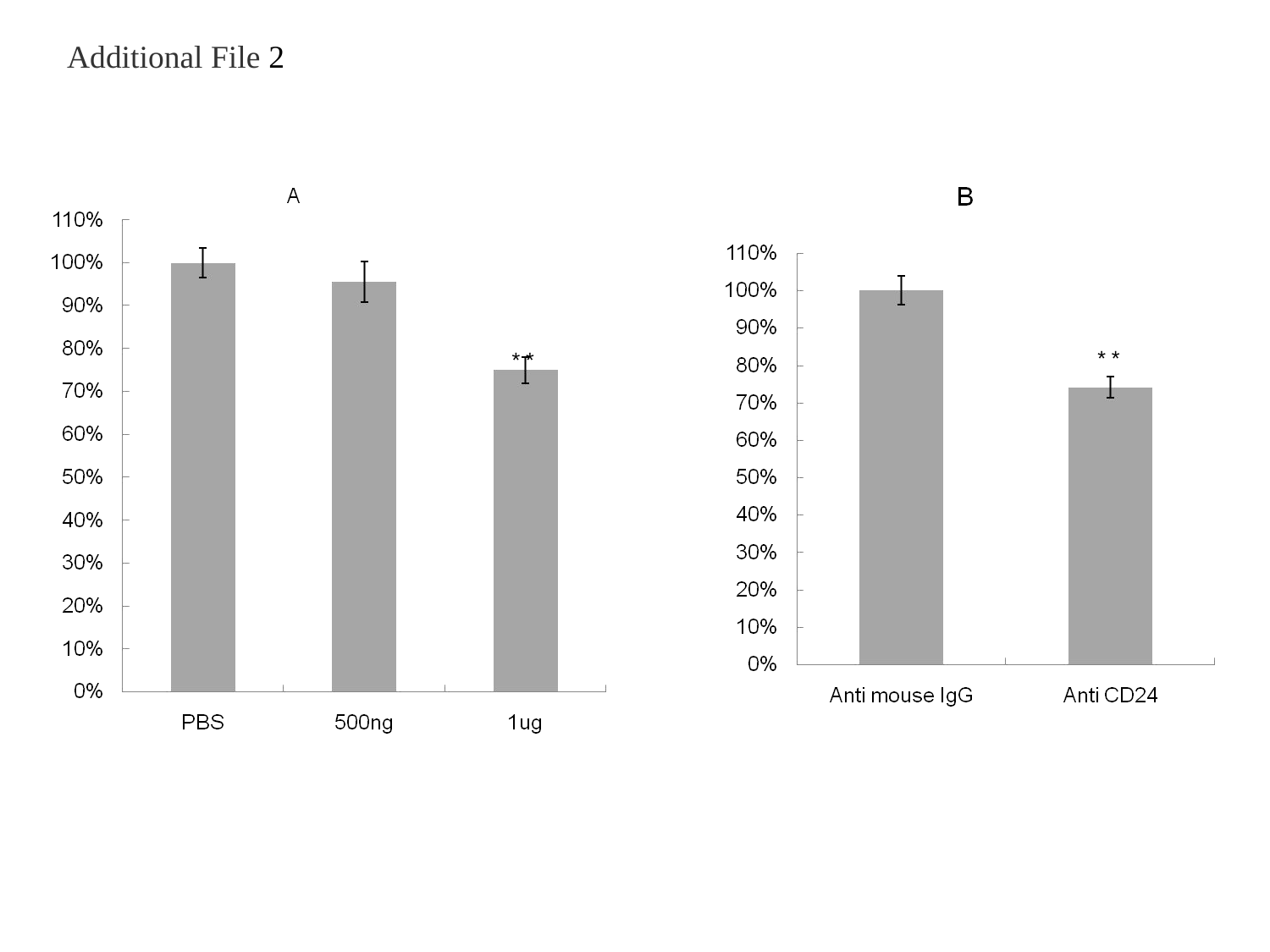

Additional File 2
* *
* *

Supplement: Additional file 2 — Viability of MCF-7 cross-linked with anti-human CD24 mouse monoclonal antibody. A) MCF-7 cells were cross-linked with anti-mouse monoclonal IgG antibody in a dose dependent manner for 72 h. B) MCF-7 cells were cross-linked with 500 ng/ml anti-mouse monoclonal IgG antibody or anti-human CD24 mouse monoclonal antibody for 72 h. A-B) Relative survival cell rate is shown as percent survivals versus in treatment versus control cells where CD24 was cross-linked with anti-mouse monoclonal IgG. Data represent means of at least three independent experiments and standard errors of the means. **, p value of less than 0.01. [file 1471-2407-8-118-S2.ppt]

## Slide 1
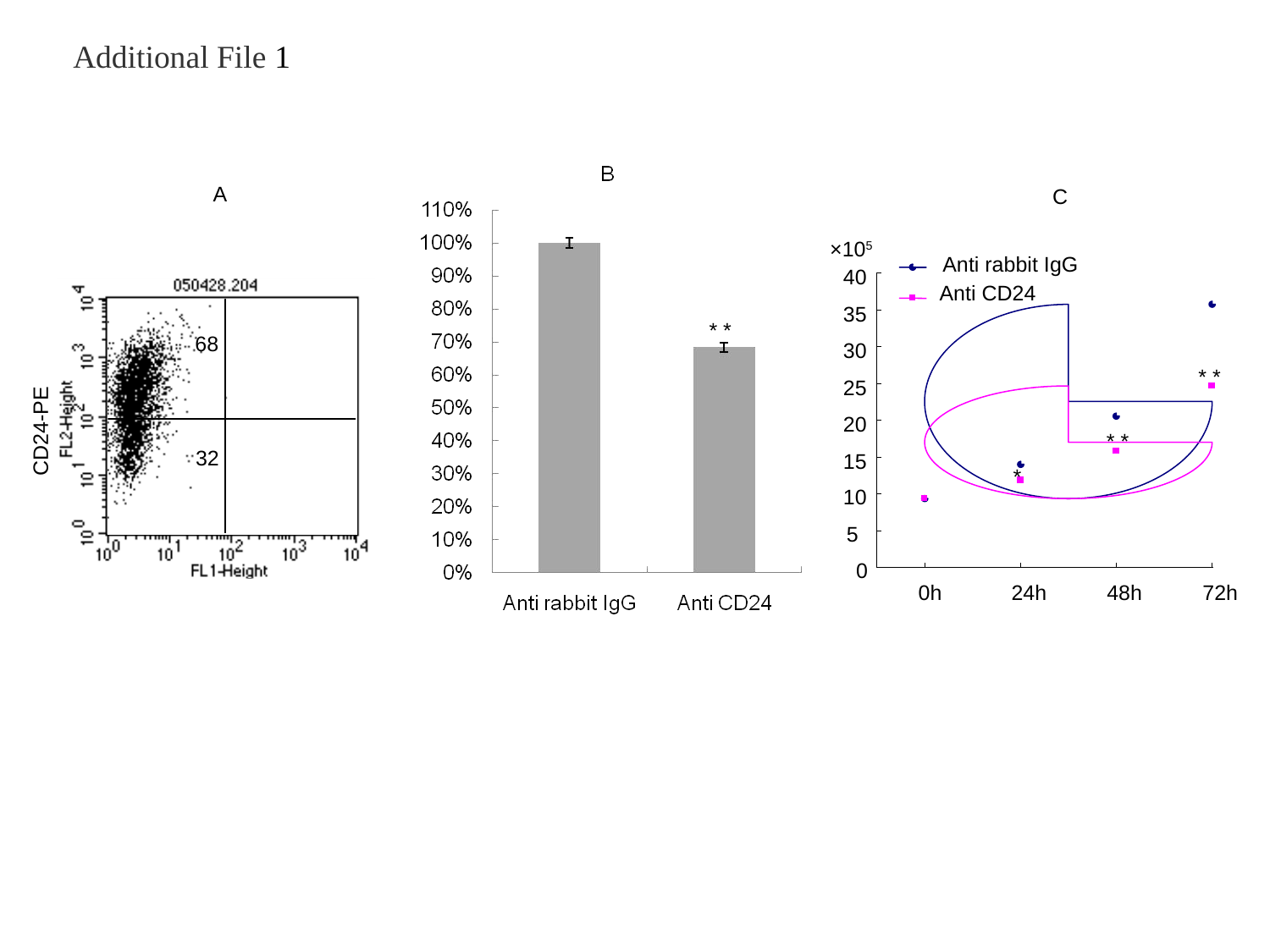

Additional File 1
A
C
×105
Anti rabbit IgG
40
Anti CD24
35
* *
68
30
* *
25
20
CD24-PE
* *
32
15
*
10
5
0
72h
0h
24h
48h

Supplement: Additional file 1 — CD24 expression and cell viability after CD24 cross-linking in MCF-10A cells. A) CD24 expression was analysed with PE anti-human CD24 antibody by flow cytometry on a FACSCalibur system. One of three the representative experiments are shown in the result. B) MCF-10A was cross-linked with 500 ng/ml anti-rabbit polyclonal IgG or anti-human CD24 rabbit polyclonal antibody 72 h. B) Relative survival cell rate is shown as percent survivals versus in treatment versus control cells where CD24 was cross-linked with anti-rabbit polyclonal IgG. C). MCF-10A was treated with 500 ng/ml of anti-rabbit polyclonal IgG or anti-human CD24 rabbit polyclonal antibody in a time dependent manner for 72 h. C) MCF-10A cell survival is shown versus control cell survival after CD24 cross-linking with anti-rabbit polyclonal IgG. Means of at least three independent experiments are presented with standard errors. *, p value of less than 0.05 **, p value of less than 0.01. [file 1471-2407-8-118-S1.ppt]
