# Supplementary material for: CD24 cross-linking induces apoptosis in, and inhibits migration of, MCF-7 breast cancer cells
Source: BMC Cancer. 2008 Apr 24;8:118. doi: 10.1186/1471-2407-8-118 (PMC2386794; doi:10.1186/1471-2407-8-118)
Supplement: Additional file 3 — Changes in apoptosis after CD24 cross-linking with anti-human CD24 mouse monoclonal antibody. MCF-7 cells were cross-linked with 500 ng/ml anti-mouse monoclonal IgG antibody or anti-human CD24 mouse monoclonal antibody for 72 h. Cells were stained with FITC-conjugated annexin V in a buffer containing propidium iodide and analyzed by flow cytometry. For each group of cells, the percentage of survival is shown in the lower left quadrant, where both in annexin V and propidium iodide levels are low. One of three representative experiments is presented. [file 1471-2407-8-118-S3.ppt]

## Slide 1
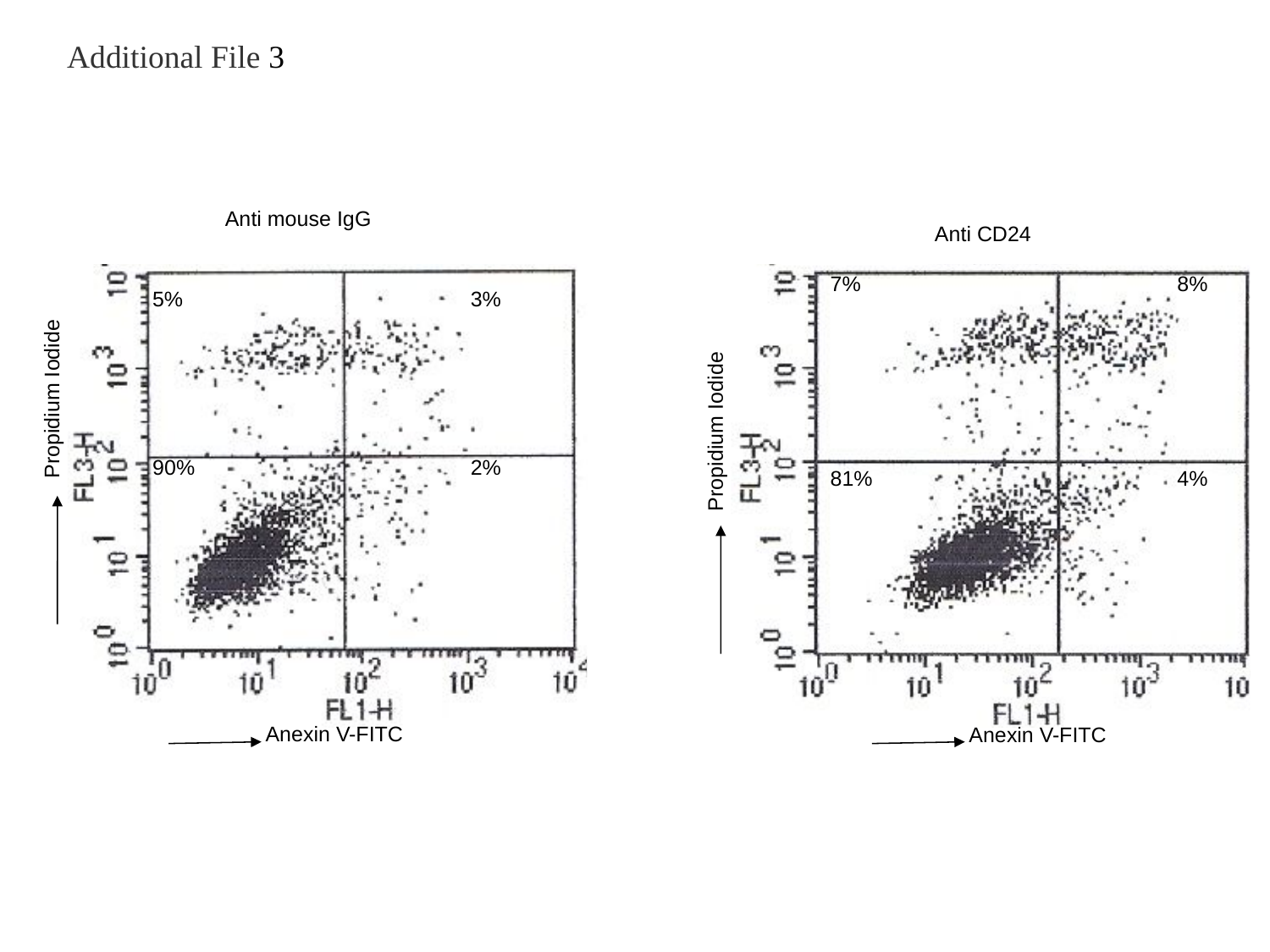

Additional File 3
Anti mouse IgG
Anti CD24
7%
8%
5%
3%
Propidium Iodide
Propidium Iodide
90%
2%
81%
4%
Anexin V-FITC
Anexin V-FITC
